# Progress in Surgery

**Published:** 1902-03-01

**Authors:** 


### Progress in Surgery.

OTOLOGY.—*Continued from page* 361.

**The Technique of Mastoid Operations.**—W. Milligan[8] alludes to the difficulty there is in the radical operation of efficiently clearing away every focus of disease. The use of burrs or osteotribes is recommended to help to decide if all disease has been removed. Before the introduction of skin grafting some parts of the tympanum after the radical operation often failed to get an epidermal covering, *e.g.* the inner tympanal wall and the receding antro-tympanic angle, and therefore a granulating surface was left. Milligan considers that Ballance's operation, which he has tried repeatedly, has revolutionised the operation for chronic otorrhœa. He suggests that the skin incision be opened up without a general anæsthetic the day before the skin grafting is performed, in order to avoid the oozing of blood during the latter procedure. Another suggestion is to fill the antro-tympanic cavity quite full of normal saline fluid, and float the graft on it, and then rapidly suck out the fluid with a pipette passed through the meatus. Thus the graft is made to sink down closely upon the walls of the cavity it is desired to cover. The writer prefers glass to metal stoppers for pressing home the gold leaf, because they adhere less to the gold leaf. The remains of the posterior meatal wall should be carefully smoothed down lest the grafts be broken there over a sharp edge. The pinna may be slung up to the top of the head by tapes soaked in collodion. Panse and Leutert[9] maintain that reinfection of the cavities operated upon in and about the ears occurs through the Eustachian tube, and they therefore endeavour to shut off the tube by causing the remnant of the membrana tympani to adhere to the promontory. Trautmann[10] states that galvano-cauterisation of the edges of the Eustachian opening in the tympanum, followed by firm tamponading against the inner tympanic wall, will effect closure. Galvano-cauterisation of the pharyngeal orifice of the tube is ineffectual. Trautmann also favours the formation of a persistent retro-auricular opening after the radical operation to bring about epidermisation. In operating he works backwards to the antrum from the attic and aditus, if he thinks the lateral sinus lies in a far forward position. This is likely to be the case if the mastoid process has a narrow and high form. If the mastoid, however, is flat and wide, the antrum may be opened at once, and the attic exposed by working forwards. He divides the cutaneous meatal canal posteriorly and horizontally, so as to form an upper narrow flap and a lower broad one. The tampons used consist of acetate of aluminium gauze or xeroform gauze. These are less irritating to the skin than iodoform gauze. Dusting the wound with orthoform renders the insertion of tampons less painful. The retro-auricular opening should not be closed for at least a year, if at all. H. Tilley[11] records a case which forcibly illustrates the tendency to meatal strain after the mastoid operation in cases where atresia exists before the operation. The patient, a woman of 65, was operated upon for pain, mastoid tenderness, and facial paralysis of acute onset, following a recurring ear discharge of 40 years' duration. In addition there was such atresia that the meatus would not admit the finest probe, the obstruction being mainly due to a large, long exostosis situated posteriorly. To try and avoid atresia a large amount of the concha was cut away. The index finger could easily be introduced into the bony cavity at the end of the operation. Five months later almost complete atresia was again present. The writer has had one other case of this complication. He alludes to Ballance's recommendation that the narrow part of the meatus be cut away and the posterior part of the wound skin grafted, so that the epithelium of the graft may unite with that of the cut edges of what remains of the meatal lining anteriorly. Charles H. May (New York) has published notes on a series of mastoid operations.[12] Some of these cases have no particular interest, but one affords an illustration of how quietly and with what mild symptoms inflammation may progress in the mastoid until there is extensive destruction of tissue. The operation was performed two months after the beginning of an ear trouble which followed influenza. Some redness and swelling of the mastoid appeared a few days after the onset but subsided again. At the time of operation there was persistent otorrhœa and very slight persistent tenderness over the mastoid antrum. The latter was found on exploration carious in its interior, and filled with much granulation tissue and pus. The tympanum was not touched. A cure resulted in six weeks. Another interesting case of May's was that of a boy four years of age, who had first the right mastoid opened for acute inflammation, and a month later the left one for similar disease. At the time of the first operation the child had diphtheria. A large amount of diseased bone was removed on each occasion. Recovery ensued.

**Auscultation of the Mastoid.**—J. A. H. Andrews,[13] in examining mastoid cases, places a stethoscope with a small bell over the tip of the process, and the handle of a vibrating tuning-fork in the neighbourhood of the antrum. When the mastoid cells are filled with pus or granulations, or when the density is increased from bone proliferation, the sound waves are transmitted to the ears of the examiner with more intensity, and for a longer time, than in the case of a normal mastoid.

**Otogenous Pyæmia.**—F. Kretschmann[14] relates three cases of this affection which are of interest. The first case was remarkable for the extent of the thrombosis present in the cranial sinuses. The patient was 68 years of age. The thrombosis began in the left transverse sinus, and finally extended through the torcular and involved the right transverse, inferior petrosal, and cavernous sinuses, and the upper part of the right jugular vein. The second case, a boy aged four years, was remarkable in that metastasis began in the lungs as early as the fourth day of an acute attack of otitis media. The drum and antrum showed very little change, and the author supposes that metastasis occurred by a leap and not by contiguity. In the third case, a boy of nine, endocarditis occurred with the pyæmia. The temperature varied from 96° F. to 106° F. The bony cochlea was ultimately thrown off as a sequestrum. The boy recovered. The tuning-fork could be heard through the bone even after this.

**Otitic Temporo-Sphenoidal Abscess.** — Dundas Grant[15] records the case of a girl aged 18. The most marked symptoms on admission were intense headache and mental disturbance, resembling acute alcoholism. The presence of chewing and swallowing movements suggested that an abscess, if present, was cerebellar. The cerebellum was first explored, with negative result. Then the temporo-sphenoidal lobe above the meatus was explored and an ounce of extremely offensive pus evacuated from it, and a drainage tube inserted. This was gradually shortened. Recovery ensued. The patient was childish for a time, but is now natural in manner again. The radical mastoid operation was done the day before the cerebral abscess was opened.

[8] Brit. Med. Jour., Sept. 28, 1901, p. 893. [9] Abst. Amer. Jour. Med. Sci., Sept. 1901, p. 369. [10] Abst. Amer. Jour. Med. Sci., July 1901, p. 121. [11] Jour. L. R. & O., Oct. 1901, p. 593. [12] Med. Record, Aug. 24, 1901, p. 290. [13] Abst. Med. Record, Aug. 3, 1901, p. 176. [14] Abst. Amer. Jour. Med. Sci., Sept. 1901, p. 369. [15] Jour. L. R. & O., Oct. 1901, p. 544.

